# Supplementary figures and images for: Staphylococcus aureus and methicillin-resistant Staphylococcus aureus in juvenile green turtle (Chelonia mydas) carcasses, rearing seawater, feed and their antibiotic resistances
Source: PeerJ. 2025 Jun 20;13:e19579. doi: 10.7717/peerj.19579 (PMC12184670; doi:10.7717/peerj.19579)

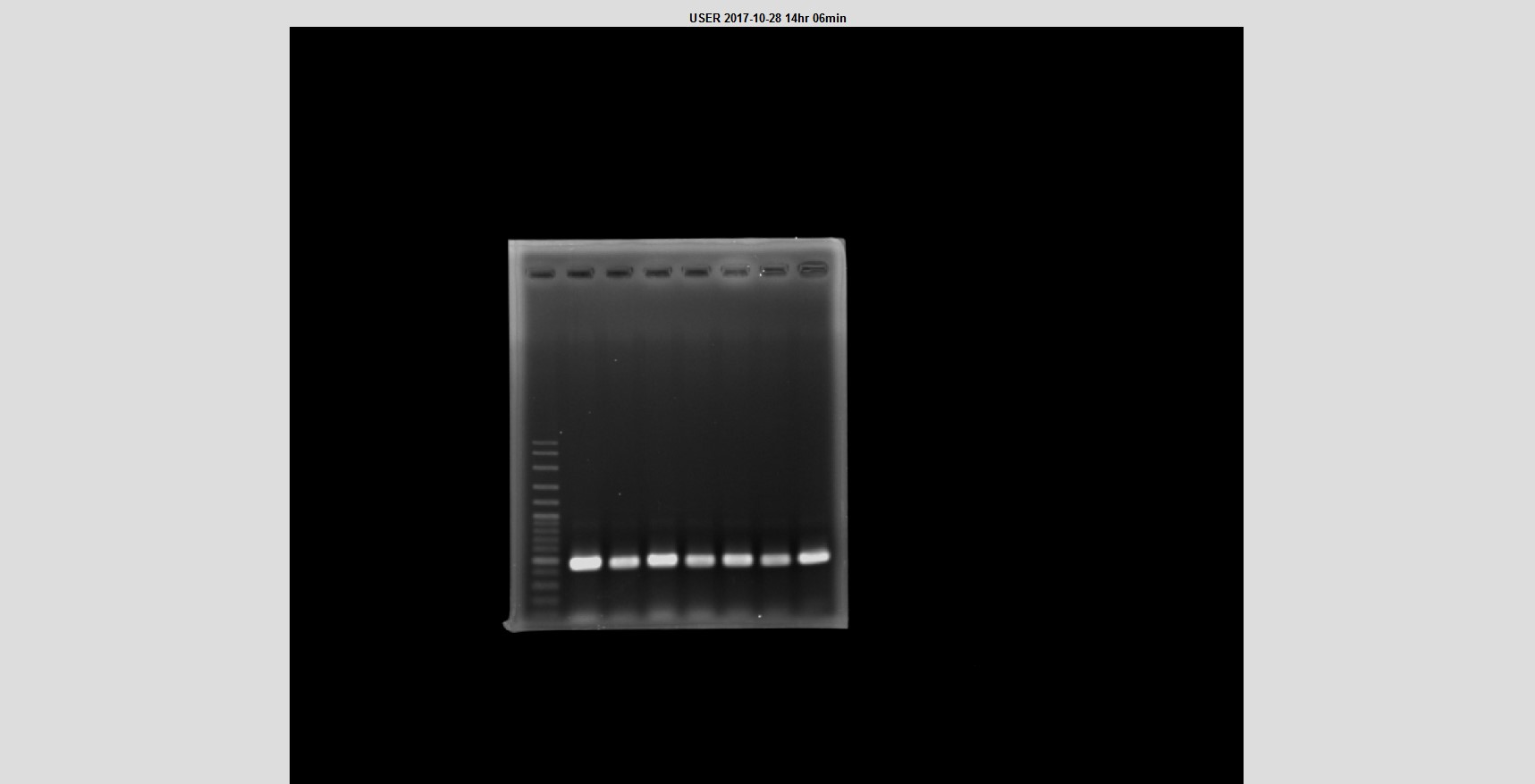

Supplement: Supplemental Information 1 [file peerj-13-19579-s001.jpg]

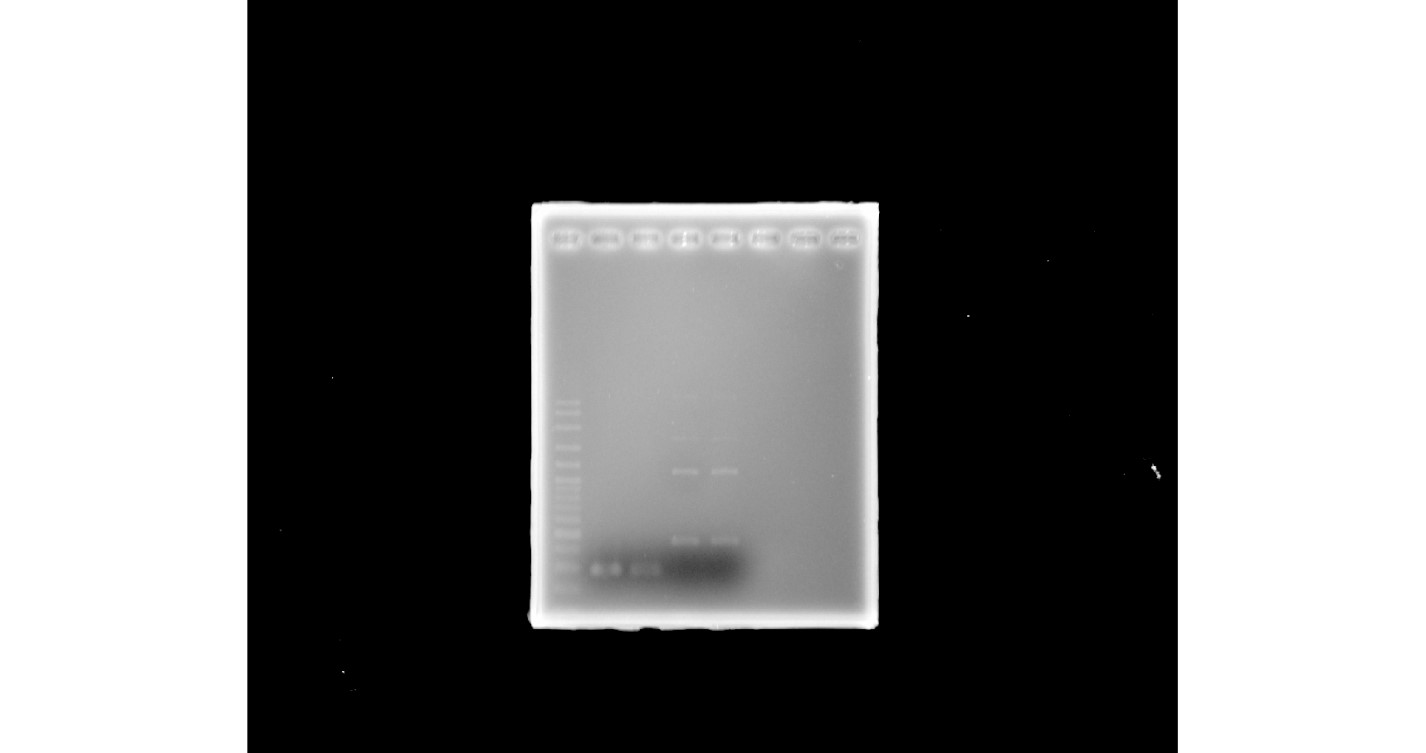

Supplement: Supplemental Information 2 [file peerj-13-19579-s002.jpg]

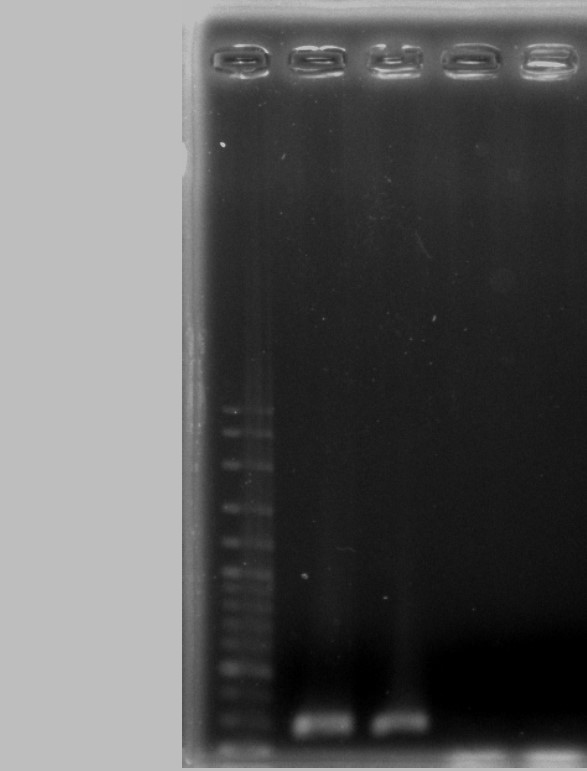

Supplement: Supplemental Information 3 [file peerj-13-19579-s003.jpg]

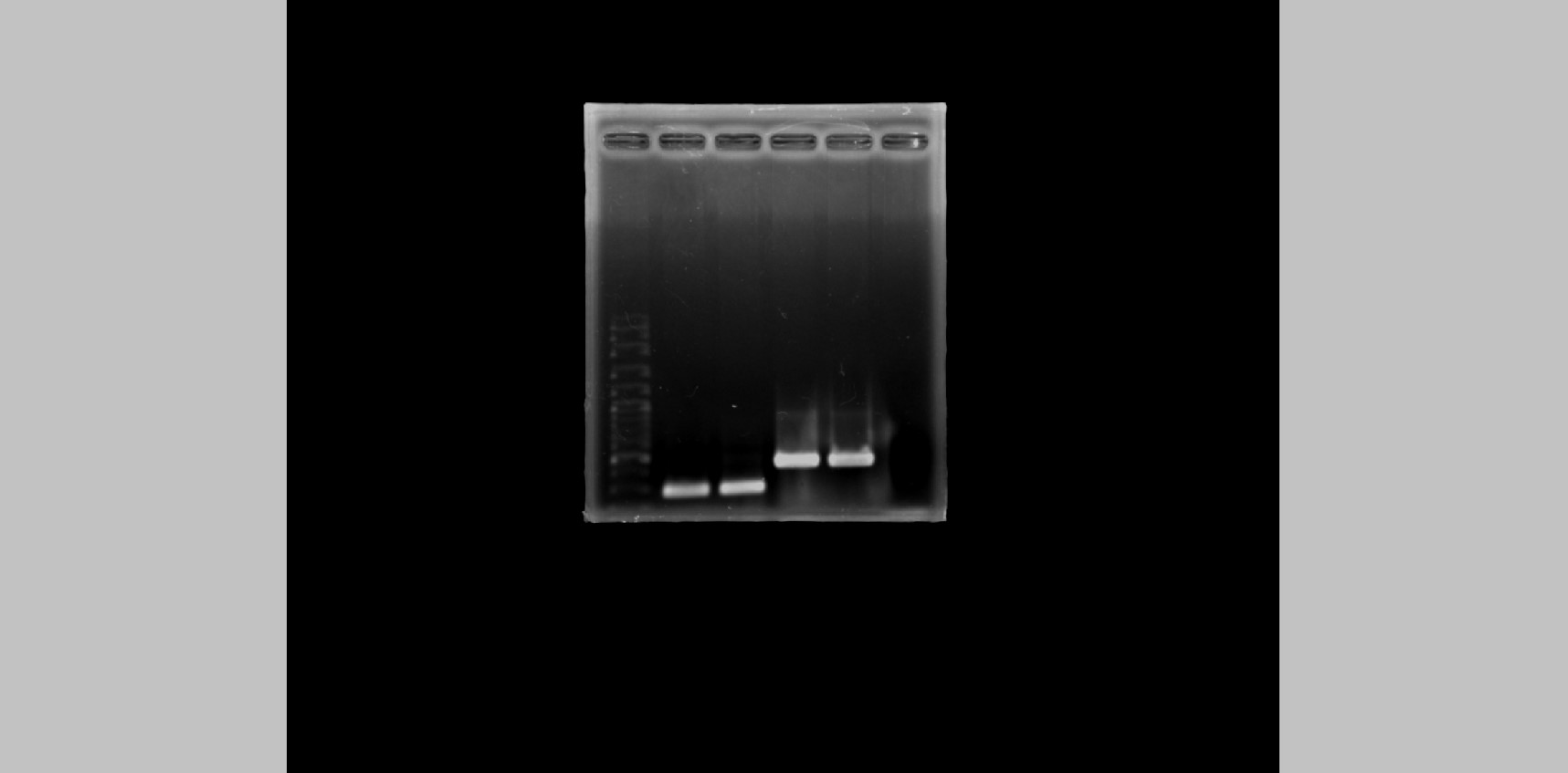

Supplement: Supplemental Information 4 [file peerj-13-19579-s004.jpg]
