# Supplementary material for: Staphylococcus aureus and methicillin-resistant Staphylococcus aureus in juvenile green turtle (Chelonia mydas) carcasses, rearing seawater, feed and their antibiotic resistances
Source: PeerJ. 2025 Jun 20;13:e19579. doi: 10.7717/peerj.19579 (PMC12184670; doi:10.7717/peerj.19579)

*mecA* gene from Staphylococcus aureus TN/CN/1/12

NCBI Reference Sequence: NG_047945.1

>NG_047945.1 *Staphylococcus aureus* TN/CN/1/12 mecA gene for ceftaroline-resistant PBP2a family peptidoglycan transpeptidase MecA, complete CDS

```
ATGAAAAAGATAAAAATTGTTCCACTTATTTTAATAGTTGTAGTTGTCGGGTTTGGTATATATT
TTTATGCTTCAAAAGATAAAGAAATTAATAATACTATTGATGCAATTGAAGATAAAAATTTCAA
ACAAGTTTATAAAGATAGCAGTTATATTTCTAAAAGCGATAATGGTGAAGTAGAAATGACTGAA
CGTCCGATAAAAATATATAATAGTTTAGGCGTTAAAGATATAAACATTCAGGATCGTAAAATAA
AAAAAGTATCTAAAAATAAAAAACGAGTAGATGCTCAATATAAAATTAAAACAAACTACGGTAA
CATTGATCGCAACGTTCAATTTAATTTTGTTAAAGAAGATGGTATGTGGAAGTTAGATTGGGAT
CATAGCGTCATTATTCCAGGAATGCAGAAAGACCAAAGCATACATATTGAAAATTTAAAATCAG
AACGTGGTAAAATTTTAGACCGAAACAATGTGGAATTGGCCAATACAGGAACAGCATATGAGAT
AGGCATCGTTCCAAAGAATGTATCTAAAAAAGATTATAAAGCAATCGCTAAAGAACTAAGTATT
TCTGAAGACTATATCAAACAACAAATGGATCAAAATTGGGTACAAGATGATACCTTCGTTCCAC
TTAAAACCGTTAAAAAAATGGATGAATATTTAAGTGATTTCGCAAAAAAATTTCATCTTACAAC
TAATGAAACAAAAAGTCGTAACTATCCTCTAGAAAAAGCGACTTCACATCTATTAGGTTATGTT
GGTCCCATTAACTCTGAAGAATTAAAACAAAAAGAATATAAAGGCTATAAAGATGATGCAGTTA
TTGGTAAAAAGGGACTCGAAAAACTTTACGATAAAAAGCTCCAACATGAAGATGGCTATCGTGT
CACAATCGTTGACGATAATAGCAATACAATCGCACATACATTAATAGAGAAAAAGAAAAAAGAT
GGCAAAGATATTCAACTAACTATTGATGCTAAAGTTCAAAAGAGTATTTATAACAACATGAAAA
ATGATTATGGCTCAGGTACTGCTATCCACCCTCAAACAGGTGAATTATTAGCACTTGTAAGCAC
ACCTTCATATGACGTCTATCCATTTATGTATGGCATGAGTAACGAAGAATATAATAAATTAACC
GAAGATAAAAAAGAACCTCTGCTCAACAAGTTCCAGATTACAACTTCACCAGGTTCAACTCAAA
AAATATTAACAGCAATGATTGGGTTAAATAACAAAACATTAGACGATAAAACAAGTTATAAAAT
CGATGGTAAAGGTTGGCAAAAAGATAAATCTTGGGGTGGTTACAACGTTACAAGATATGAAGTG
GTAAATGGTAATATCGACTTAAAACAAGCAATAGAATCATCAGATAACATTTTCTTTGCTAGAG
TAGCACTCGAATTAGGCAGTAAGAAATTTGAAAAAGGCATGAAAAAACTAGGTGTTGGTGAAGA
TATACCAAGTGATTATCCATTTTATAATGCTCAAATTTCAAACAAAAATTTAGATAATGAAATA
TTATTAGCTGATTCAGGTTACGGACAAGGTGAAATACTGATTAACCCAGTACAGATCCTTTCAA
TCTATAGCGCATTAGAAAATAATGGCAATATTAACGCACCTCACTTATTAAAAGACACGAAAAA
CAAAGTTTGGAAGAAAAATATTATTTCCAAAGAAAATATCAATCTATTAACTGATGGTATGCAA
CAAGTCGTAAATAAAACACATAAAGAAGATATTTATAGATCTTATGCAAACTTAATTGGCAAAT
CCGGTACTGCAGAACTCAAAATGAAACAAGGAGAAACTGGCAGACAAATTGGGTGGTTTATATC
ATATGATAAAGATAATCCAAACATGATGATGGCTATTAATGTTAAAGATGTACAAGATAAAGGA
ATGGCTAGCTACAATGCCAAAATCTCAGGTAAAGTGTATGATGAGCTATATGAGAACGGTAATA
AAAAATACGATATAGATGAATAACAAAACAGTGAAGCAATCCGTAACGATGGTTGCTTCACTGT
TTTATTATGAATTATTAATAAGTGCTGTTACTTCTCCCTTAAATACAATTTCTTCATTT
2107 bp
```

DNA sequences from PCR product using mecA-specific primers and genomic DNA of isolate from this study

>3.7

TACGAGTAGATGCTCAATATAAAATTAAAACAAACTACGGTAACATTGATCGCAACGTTCAATT
TAATTTTGTTAAAGAAGATGGTATGTGGAAGTTAGATTGGGATCATAGCGTCATTATTCCAGGA
ATGCAGAAAGACCAAAGCATACATATTGAAAATTTAAAATCAGAACGTGGTAAAATTTTAGACC
GAAACAATGTGGAATTGGCCAATACAGGAACAGCATATGAGATAGGCATCGTTCCAAAGAATGT
ATCTAAAAAAGATTATAAAGCAATCGCTAAAGAACTAAGA 296 bp

>4.8

GAGTAGATGCTCAATATAAAATTAAAACAAACTACGGTAACATTGATCGCAACGTTCAATTTAA
TTTTGTTAAAGAAGATGGTATGTGGAAGTTAGATTGGGATCATAGCGTCATTATTCCAGGAATG
CAGAAAGACCAAAGCATACATATTGAAAATTTAAAATCAGAACGTGGTAAAATTTTAGACCGAA
ACAATGTGGAATTGGCCAATACAGGAACAGCATATGAGATAGGCATCGTTCCAAAGAATGTATC
TAAAAAAGATTATAAAGCAATCGCTAAAGAACTAAGA 293 bp

Results from aliment of DNA sequences with NCBI Reference Sequence: NG_047945.1

| Description | Max score | Total score | Query score | E value | Per. Ident | Acc. Len |
|---|---|---|---|---|---|---|
| 3.7 | 544 | 544 | 14% | 1e-158 | 100.00% | 296 |
| 4.8 | 540 | 540 | 14% | 1e-157 | 100.00% | 293 |

3.7

```
Query  279   acgagtagatgctcaatataaaattaaaacaaaCTACGGTAACATTGATCGCAACGTTCA  338
             ||||||||||||||||||||||||||||||||||||||||||||||||||||||||||||
Sbjct  2     ACGAGTAGATGCTCAATATAAAATTAAAACAAACTACGGTAACATTGATCGCAACGTTCA  61

Query  339   ATTTAATTTTGTTAAAGAAGATGGTATGTGGAAGTTAGATTGGGATCATAGCGTCATTAT  398
             ||||||||||||||||||||||||||||||||||||||||||||||||||||||||||||
Sbjct  62    ATTTAATTTTGTTAAAGAAGATGGTATGTGGAAGTTAGATTGGGATCATAGCGTCATTAT  121

Query  399   TCCAGGAATGCAGAAAGACCAAAGCATACATATTGAAAATTTAAAATCAGAACGTGGTAA  458
             ||||||||||||||||||||||||||||||||||||||||||||||||||||||||||||
Sbjct  122   TCCAGGAATGCAGAAAGACCAAAGCATACATATTGAAAATTTAAAATCAGAACGTGGTAA  181

Query  459   AATTTTAGACCGAAACAATGTGGAATTGGCCAATACAGGAACAGCATATGAGATAGGCAT  518
             ||||||||||||||||||||||||||||||||||||||||||||||||||||||||||||
Sbjct  182   AATTTTAGACCGAAACAATGTGGAATTGGCCAATACAGGAACAGCATATGAGATAGGCAT  241

Query  519   CGTTCCAAAGAATGTATCTAAAAAAGATTATAAAGCAATCGCTAAAGAACTAAG  572
             ||||||||||||||||||||||||||||||||||||||||||||||||||||||
Sbjct  242   CGTTCCAAAGAATGTATCTAAAAAAGATTATAAAGCAATCGCTAAAGAACTAAG  295
```

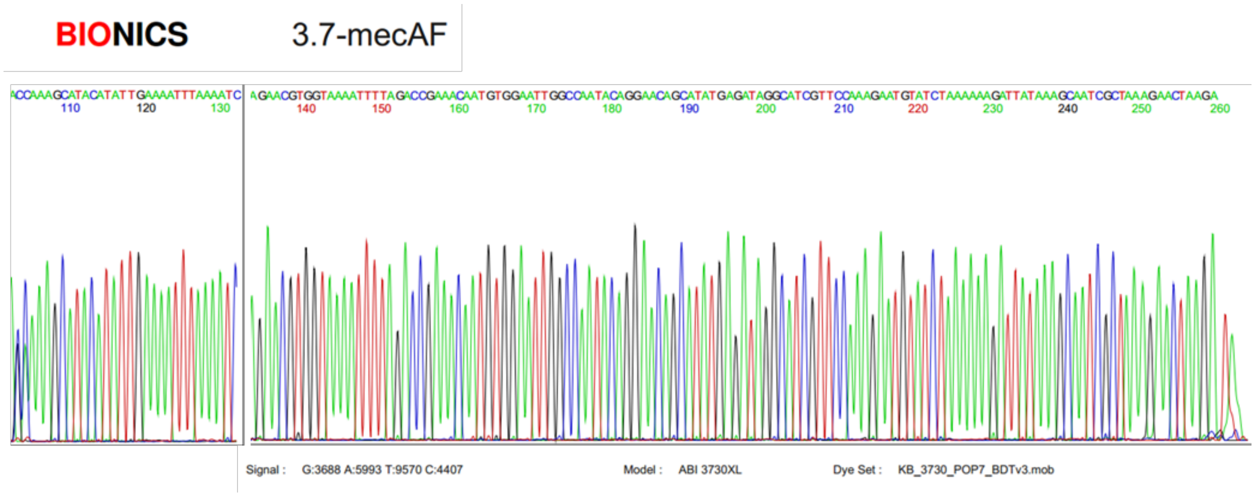

4.8

```
Query  281   gagtagatgctcaatataaaattaaaacaaaCTACGGTAACATTGATCGCAACGTTCAAT  340
             ||||||||||||||||||||||||||||||||||||||||||||||||||||||||||||
Sbjct  1     GAGTAGATGCTCAATATAAAATTAAAACAAACTACGGTAACATTGATCGCAACGTTCAAT  60

Query  341   TTAATTTTGTTAAAGAAGATGGTATGTGGAAGTTAGATTGGGATCATAGCGTCATTATTC  400
             ||||||||||||||||||||||||||||||||||||||||||||||||||||||||||||
Sbjct  61    TTAATTTTGTTAAAGAAGATGGTATGTGGAAGTTAGATTGGGATCATAGCGTCATTATTC  120

Query  401   CAGGAATGCAGAAAGACCAAAGCATACATATTGAAAATTTAAAATCAGAACGTGGTAAAA  460
             ||||||||||||||||||||||||||||||||||||||||||||||||||||||||||||
Sbjct  121   CAGGAATGCAGAAAGACCAAAGCATACATATTGAAAATTTAAAATCAGAACGTGGTAAAA  180

Query  461   TTTTAGACCGAAACAATGTGGAATTGGCCAATACAGGAACAGCATATGAGATAGGCATCG  520
             ||||||||||||||||||||||||||||||||||||||||||||||||||||||||||||
Sbjct  181   TTTTAGACCGAAACAATGTGGAATTGGCCAATACAGGAACAGCATATGAGATAGGCATCG  240

Query  521   TTCCAAAGAATGTATCTAAAAAAGATTATAAAGCAATCGCTAAAGAACTAAG  572
             ||||||||||||||||||||||||||||||||||||||||||||||||||||
Sbjct  241   TTCCAAAGAATGTATCTAAAAAAGATTATAAAGCAATCGCTAAAGAACTAAG  292
```

**BIONICS** 4.8-mecAF

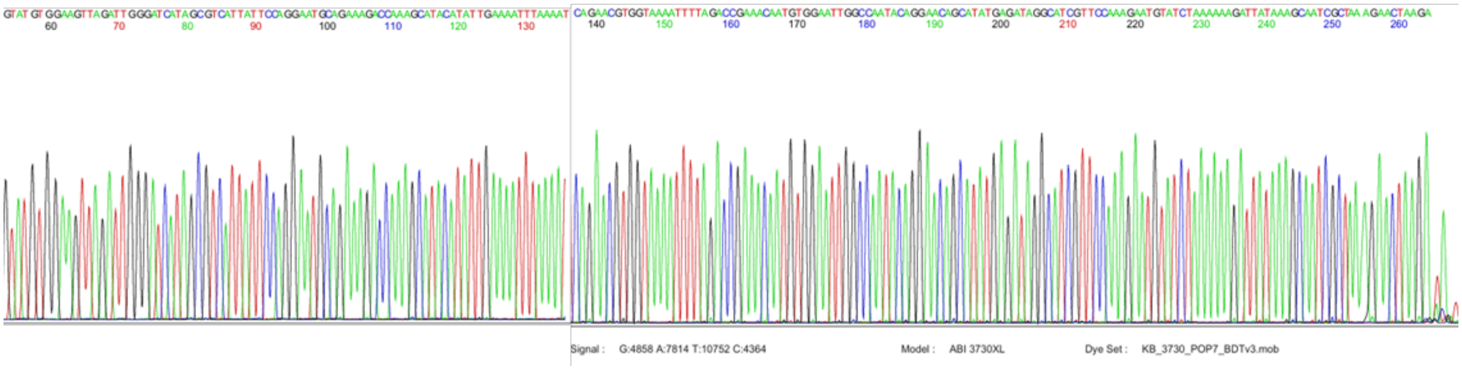

Supplement: Supplemental Information 6 — Sequencing chromatograms of PCR product using mecA-specific primers and alignment of amplicon sequnences and mecA gene. [file peerj-13-19579-s006.pdf]
